# The Study of Medicine

**Published:** 1905-09-02

**Authors:** 


### The Hospital.

A Journal of

The Medical Sciences and Hospital Administration

Vol. XXXVIII.—No. 988. SATURDAY, SEPTEMBER 2, 1905.

### The Study of Medicine.

With the commencement of the coming session of the medical schools, a new era of teaching will be inaugurated in the metropolis; and it is not too much to say that this new era will have as its chief characteristic an endeavour to place the study of medicine upon a basis more truly scientific than that upon which it has hitherto rested, and to restrain the element of empiricism, using the word in its proper sense, within the narrowest limits which circumstances will allow. The University of London, acting in the first instance through two of its colleges, King's College and University College, has made arrangements for the conduct of the preliminary anatomical, scientific, and laboratory teaching of students whose subsequent clinical education will be conducted elsewhere; and will in this way be enabled, as time goes on, to furnish the metropolitan hospitals with a constant supply of men already well grounded in anatomy and physiology, and prepared, by a preliminary study of the methods and results of physical science, to take part in the observation and investigation of vital actions and phenomena, and of the manner in which these actions and phenomena may be modified by what we call disease. St. George's Hospital and the Westminster Hospital have already, we understand, definitely abandoned the "preliminary" teaching which has hitherto been conducted in their respective schools, and have made arrangements by which the earlier portions of the official curriculum will be relegated to one or other of the above-named colleges. The example thus set must, we believe, be universally followed before long; for, although the authorities of some of the London schools are said to be still recalcitrant, or at least prepared to maintain their complete sufficiency for all requirements, it can hardly be doubted that the status, and ultimately, we hope, the emoluments of the University Professors will command the services of men of the first rank in their several departments of science, and that these, in their turn, will attract the great majority of really earnest and capable students to their class-rooms. It is impossible to deny that, speaking generally, the so-called preliminary instruction hitherto given at the London medical schools has been given by men whose own hopes and aspirations have rested rather upon the application than upon the communication of knowledge; that the teacher of anatomy, for example, has usually been looking forward to the time when his acquirements would be displayed chiefly in the operating theatre, and the teacher of physiology to the time when he would attain the goal of his ambitions as a physician in the wards. The work of teaching, in too many instances, has been regarded mainly as a condition of preferment; and, in these circumstances, it can hardly have failed to lose something of the earnestness and the force which would have been infused into it by a professor to whom it would represent the primary duty of his life, and the chief end to which his energies should be directed.

Among the other unfortunate consequences of this method of procedure, more especially in relation to physical science, we should be inclined to place what we regard as a very imperfect appreciation of its importance, as a basis of medicine, on the part of some of those who occupy positions of prominence and of responsibility in the profession. Medicine is essentially the study of the relations between man and his environment, and of the methods in which those relations may be modified either by nature or by art. A large proportion of what we call diseases are only the symptomatic expressions of perversions of the chemical processes of metabolism, and can neither be understood nor rationally treated by practitioners to whom organic chemistry is either a sealed book, or even a verbal collection of the hypotheses of two or three years ago. Another enormous proportion are only the symptomatic expressions of struggles for survival between man and inferior organisms, and require for their due comprehension and proper treatment, a knowledge of biology of a far more accurate and comprehensive kind than is generally either possessed, or even recognised to be necessary.

It is not enough, for the status and for the usefulness of the profession, that a few of its members should be men of science in the highest sense of the term, so long as a majority aim at nothing more than the empirical application of knowledge which has, indeed, a scientific basis, but a basis which they are not able either thoroughly to understand or clearly to explain. The public are not very wise; and it is possible that even Carlyle's estimate of their aggregate capacity might be accepted without any very serious modification.

Notwithstanding this, they will generally be found to have a more or less hazy recognition of the presence of superior knowledge, and a corresponding readiness to be guided by it when once they believe that it is there. The kinsfolk of medical students form a large aggregate of persons; and these, if they are once made to believe that the training of their young friends has a real foundation in science, as distinguished from experience and tradition, will be likely to exert a potent influence upon the public opinion of the future, and thus to secure, for the professors of medicine, a larger and more useful amount of influence than it has even hitherto been their lot to possess.

